# The level of manganese superoxide dismutase content is an independent prognostic factor for glioblastoma. Biological mechanisms and clinical implications

**DOI:** 10.1054/bjoc.2000.1594

**Published:** 2001-02

**Authors:** F Ria, M Landriscina, F Remiddi, R Rosselli, M Iacoangeli, M Scerrati, G Pani, S Borrello, T Galeotti

**Affiliations:** Institute of General Pathology and; Institute of Neurosurgery, Catholic University, L. go F. Vito, Rome, 1. I-00168, Italy

**Keywords:** MnSOD, p53, glioblastoma, reactive oxygen species

## Abstract

We address the issue of the role of manganese superoxide dismutase in tumorigenesis by studying a relatively homogeneous group of tumours for the correlation between amount of this anti-oxidant enzyme and prognosis. The clinical outcome of 30 patients affected by glioblastomas whose manganese superoxide dismutase content had been established at the time of first diagnosis is compared. When the survival of patients is stratified according to manganese superoxide dismutase level in the tumour, a link of these levels and prognosis can be observed. Patients with high levels of manganese superoxide dismutase show a median survival time of 6.11 months, while patients whose tumours display a low amount of MnSOD have a median survival time of 12.17 months. To assess the upstream mechanisms that sustain the increase in manganese superoxide dismutase content in brain neuroepithelial tumours, we also studied the expression of p53 in a series of 17 astrocytomas of various grading. In all tested astrocytomas, high manganese superoxide dismutase content is associated with cytoplasmic accumulation of p53. Thus glioblastomas can be divided into two distinct groups on the basis of their content of manganese superoxide dismutase, having ‘better’ or ‘worse’ prognosis, respectively. The use of this protein as a marker may help to define therapeutic strategies in the clinical management of glioblastoma. http://www.bjcancer.com © 2001 Cancer Research Campaign


*British Journal of Cancer* (2001) **84**(4), 529–534
© 2001 Cancer Research Campaign
doi: 10.1054/ bjoc.2000.1594, available online at http://www.idealibrary.com on IDEAL®

http://www.bjcancer.com

### The level of manganese superoxide dismutase content is an independent prognostic factor for glioblastoma. Biological mechanisms and clinical implications

**F Ria,[1] M Landriscina,[1] F Remiddi,[1] R Rosselli[2], M Iacoangeli[2], M Scerrati[2], G Pani[1], S Borrello[1] and T Galeotti[1]**

[1]Institute of General Pathology and [2]Institute of Neurosurgery, Catholic University, L. go F. Vito, 1. I-00168 Rome, Italy

**Summary** We address the issue of the role of manganese superoxide dismutase in tumorigenesis by studying a relatively homogeneous group of tumours for the correlation between amount of this anti-oxidant enzyme and prognosis. The clinical outcome of 30 patients affected by glioblastomas whose manganese superoxide dismutase content had been established at the time of first diagnosis is compared. When the survival of patients is stratified according to manganese superoxide dismutase level in the tumour, a link of these levels and prognosis can be observed. Patients with high levels of manganese superoxide dismutase show a median survival time of 6.11 months, while patients whose tumours display a low amount of MnSOD have a median survival time of 12.17 months. To assess the upstream mechanisms that sustain the increase in manganese superoxide dismutase content in brain neuroepithelial tumours, we also studied the expression of p53 in a series of 17 astrocytomas of various grading. In all tested astrocytomas, high manganese superoxide dismutase content is associated with cytoplasmic accumulation of p53. Thus glioblastomas can be divided into two distinct groups on the basis of their content of manganese superoxide dismutase, having ‘better’ or ‘worse’ prognosis, respectively. The use of this protein as a marker may help to define therapeutic strategies in the clinical management of glioblastoma. © 2001 Cancer Research Campaign http://www.bjcancer.com

**Keywords:** MnSOD; p53; glioblastoma; reactive oxygen species

Superoxide dismutases (SODs) are oxy-radical scavenger enzymes that catalyse the dismutation of $O^-_2$ to $H_2O_2$ and whose decrease has been related to tumorigenesis in several experimental models and human cancers (e.g. see Galeotti et al, 1989; Sun, 1990).

Over-production of reactive oxygen species (ROS) has been associated with DNA damage and tumour induction (Okada et al, 1999). The hypothesis of a role for such a pathogenic mechanism has been reinforced by the observation that several oxy-radical scavenger enzymes decrease during tumour progression, and that less differentiated tumours usually have lower anti-oxidant capacity. According to this hypothesis, it has been reported that manganese-dependent SOD (MnSOD) over-expression in several cell types (SV40 transformed fibroblast,(Bravard et al, 1992a), melanoma (Bravard et al, 1999), prostate cancer cells (Li et al, 1998) could restore a ‘normal’ phenotype, and it was suggested that this type of SOD could be considered as an anti-oncogene (Bravard et al, 1992b).

In contrast with this view, an increase in levels of MnSOD has been reported in the sera of 50% of patients suffering from ovarian cancer and neuroblastomas (Ishikawa et al, 1990; Kawamura et al, 1992). We (Landriscina et al, 1996) and others have reported that MnSOD content in human astrocytomas increases together with grading, in a statistically significant manner. Increase of MnSOD levels were also observed in pleural mesothelioma (Kahlos et al, 1998) and renal carcinoma (Sarto et al, 1999). A further support to the hypothesis that oxy-radical scavenger enzymes can be beneficial to brain tumour survival comes from a report showing that also Cu/ZnSOD increases with grading in astrocytomas (although without reaching statistical significance), and it apparently confers resistance to drug- and radiotherapy to the tumour itself (Yoshii et al, 1999). Reduction of sensitivity to radiation therapy has also been implied as the mechanism that sustains the role as negative prognostic factor of MnSOD for local recurrence of cervical carcinomas (Nakano et al, 1996). The possibility that an increase of MnSOD level is a relatively late event that boosts the malignant potency of tumours was already suggested by our data and is further supported by the work of Lam et al (1999) showing that over-expression of MnSOD results in increase of cell invasiveness in hamster cheek pouch carcinoma cells, and by data reported by Janssen et al (1999), showing that neoplastic epithelial cells and metastasis of colon carcinomas had levels of this enzyme higher than intermediate dysplastic adenoma cells.

The hypothesis that MnSOD may act as an ‘oncogene’ is also sustained by several recent data that suggest a model in which oxy-radicals play a relevant role in the induction of apoptosis (e.g. see Amoroso et al, 1999; Jayanthi et al, 1999). Several reports have highlighted the role that mitochondrial and redox-related enzymes may play in the induction of apoptosis and, conversely, in the escape from apoptosis during tumour induction and progression. As an example, an efflux of cytochrome *c* from mitochondria has been demonstrated to be a major event in apoptosis (for a review see Green and Reed, 1998). MnSOD has been suggested to play a relevant role in protecting hippocampal neurons against oxidative stress-induced apoptosis (Mattson et al, 1997), and it has been shown to protect cells from mitochondrial initiated cell death (Kinningham et al, 1999).

A helpful piece of information about the role that oxy-radical scavenger enzymes play in tumours would be to relate their levels

*Received 27 March 2000*
*Revised 31 July 2000*
*Accepted 16 October 2000*

*Correspondence to:* T Galeotti to prognosis. In an ideal setting, one would hope to have tumours of the same histological type and of the same grading that differ in the content of a given enzyme. Were this enzyme favourable for tumour growth (such as it would be if it is involved in protection from induction of apoptosis), one would expect to have a worse prognosis for those tumours that show higher levels of enzyme. The opposite would be expected in the case that the effect of the enzyme is detrimental for tumour progression (such as if it mainly removes agents favouring cell proliferation or tumour progression). In our previous work (Landriscina et al, 1996) we reported that glioblastomas showed a bimodal distribution of their MnSOD content, and could therefore be divided in two groups, characterized by lower or higher amounts of MnSOD, and encompassing approximately 30% and 70% of total glioblastomas, respectively. Therefore in this tumour model, we are in the condition to evaluate the potential benefit of using the MnSOD content as a prognostic factor for this subset of brain neuroepithelial tumours. In the present paper we compare the clinical outcome of 30 patients bearing glioblastomas whose MnSOD content had been established at the time of the first diagnosis. According to the criteria reported in (Landriscina et al, 1996) patients were divided in two groups, low MnSOD (for MnSOD content in class I and II, L.S.), and high MnSOD (for an MnSOD content in class III or IV, H.S.), and the survival curves were matched. As a further practical outcome, the results of this experiment may be applied to the evaluation of low grade astrocytomas, among which approximately 10% show an accelerated course.

A further point that needs to be clarified is which biological events result in MnSOD up-regulation in brain tumours. MnSOD is an inducible enzyme, whose up-regulation in response to ROS or LPS (possibly via TNF) is controlled by AP-1 (which is, in turn, under the regulation of MAP kinases signalling pathway) (Borrello and Demple, 1997). Recent reports have also highlighted the role of NF-kappa B in its positive regulation (Mattson et al, 1997; Xu et al, 1999). However, we have also observed that inactivation of p53 function results in over-expression of MnSOD (Pani et al, 2000), and it has been reported that expression of wild-type p53 could inhibit the induction of MnSOD by TNF (Shatrov et al, 2000). Furthermore, a statistical association between high MnSOD levels and nuclear accumulation of p53 was observed in cervical cancer (Nakano et al, 1996). p53 plays a major role at the joint between redox balance and apoptosis and it has been reported that p53 is involved in up- or down-regulation of the expression of several genes involved in the redox balance (Polyak et al, 1997). p53 inactivation is a most frequent event in tumour progression, and it can result from several molecular mechanisms, such as binding to viral proteins, dominant inactivating mutation or deletion. Therefore, although p53 alteration had already been studied in brain tumours of neuroepithelial origin (see e.g. von Deimling et al, 1992; Rasheed et al, 1994) we decided to use this model to examine the relationship between p53 histological behaviour and high or low MnSOD level.

### MATERIALS AND METHODS

### Patients and statistical analysis

30 consecutive patients suffering from grade IV astrocytoma were admitted to our Neurosurgery division between June 1994 and June 1997. The clinical characteristics of these patients are listed in Table 1. All subjects underwent surgical ablation of the tumour

**Table 1** Clinical characteristics of glioblastoma patients

| Patient | Survival (months) | MnSOD[a] | Age (Years) | Sex | Residual disease | KPS |
|---|---|---|---|---|---|---|
| 1 | 24 | LS | 67 | M | <10% | 90 |
| 2 | 3 | HS | 46 | M | >10% | 60 |
| 3 | 17 | HS | 59 | F | >10% | 80 |
| 4 | 6 | HS | 74 | F | >10% | 70 |
| 5 | 2 | HS | 63 | F | >10% | 60 |
| 6 | 12 | LS | 53 | M | >10% | 80 |
| 7 | 12 | LS | 55 | M | >10% | 80 |
| 8 | 8 | HS | 45 | F | >10% | 60 |
| 9 | 12 | HS | 31 | M | <10% | 80 |
| 10 | 8 | LS | 57 | F | >10% | 70 |
| 11 | 8 | LS | 45 | M | >10% | 60 |
| 12 | 2 | HS | 49 | F | >10% | 70 |
| 13 | 12 | LS | 57 | F | <10% | 60 |
| 14 | 3 | HS | 59 | M | >10% | 60 |
| 15 | 1 | HS | 75 | F | >10% | 60 |
| 16 | 11 | LS | 62 | F | <10% | 80 |
| 17 | 7 | HS | 40 | M | >10% | 60 |
| 18 | 4 | HS | 50 | M | >10% | 70 |
| 19 | 2 | HS | 68 | F | >10% | 60 |
| 20 | 5 | HS | 60 | M | >10% | 70 |
| 21 | 13 | LS | 45 | M | <10% | 80 |
| 22 | 5 | HS | 65 | M | >10% | 60 |
| 23 | 8 | LS | 73 | F | >10% | 60 |
| 24 | 13 | HS | 53 | F | <10% | 80 |
| 25 | 8 | LS | 60 | M | >10% | 70 |
| 26 | 6 | LS | 45 | F | >10% | 60 |
| 27 | 1 | HS | 74 | F | >10% | 50 |
| 28 | 23 | LS | 54 | M | >10% | 70 |
| 29 | 6 | HS | 65 | M | >10% | 70 |
| 30 | 14 | LS | 66 | F | >10% | 70 |

[a] LS and HS are described under Material and Methods.

 © 2001 Cancer Research Campaign and intraoperative specimens were frozen soon after removal as described below, for determination of MnSOD levels. The patients were followed for up to two years for survival, and all of patients died within this time lag. 15 out of 30 patients belong to the group that was studied in our previous work (Landriscina et al, 1996).

For statistical analysis, patients were grouped according to age, sex, post-surgery residual disease, Karnofsky Performance Status, MnSOD levels, as described in detail in the Results section. Survival estimates were generated by the Kaplan-Meyer product-limit method (Kaplan and Meyer, 1958). Survival times were measured from the date of surgery to the date of death. The log-rank test was used to compare the distribution of survival times in univariate analysis and to select statistically significant factors. The multivariate analysis was performed using the Cox proportional hazard model (stepwise selection: enter limit at $P < 0.1$, remove limit $P > 0.15$) (Cox, 1972). Relative risks were expressed as the ratio of relative death rate in two groups (Oi/Ei, O: observed, E: expected). A $P$ value < 0.05 was considered as statistically significant.

### Evaluation of MnSOD levels

MnSOD content in tumour brains was evaluated using either or both immunohistology and Western blot, as described in Landriscina et al (1996), using a commercial anti-human MnSOD antiserum, purchased from Calbiochem (La Jolla, Ca, USA). Briefly, intraoperative specimens were obtained during surgical removal of the tumours and immediately frozen. 10 μm slices were cut and fixed in acetone-methanol (50% v/v), washed three times in phosphate buffered saline 0.1 M (PBS) and treated with protein blocking agent (Immunon-Lipshaw, Pittsburg, PA, USA). Slices were incubated with anti-human MnSOD antiserum or appropriate control serum diluted according to manufacturer's instruction and later with alkaline phosphatase conjugated antibody, followed by BCIP/NBT. Sequential slices were evaluated in order to confirm the histological grading of the specimen. For Western blot, homogenates of samples were immuno-precipitated and loaded on a 12.5% gel for SDS-PAGE. Gels were blotted onto nitrocellulose paper and stained for MnSOD as described (Ria et al, 1993).

The results were expressed on a semiquantitative scale in comparison with human liver (++++ in both immunohistology and Western blot) and normal glia (+ in Western blot and – in immunohistology). Intermediate values in immunohistology were given either for lower intensity of staining throughout the entire slice, or for intense staining of isolated cells or of small areas. Necrotic areas (when present) were excluded in weighing the level of MnSOD or p53. For both Western blot and immunostaining the results were scored by three independent observers and in a blind fashion with respect to histological grading. MnSOD content was divided into Low SOD (LS) for MnSOD content ranging from – to ++, and high SOD (HS) for MnSOD content evaluated as +++ to ++++. Detailed examples for both Western blot and immunohistology are shown in our previous work (Landriscina et al, 1996).

### Immunohistology for p53

Between February 1996 and June 1997, 4 samples from grade II astrocytomas, 4 from grade III astrocytomas and 9 from glioblastomas were also collected for immunostaining for p53 and MnSOD. Tumour slices adjacent to those prepared for MnSOD were fixed following the same protocol described above and matched with mouse anti-human p53 antibody DO1 or isotype matched antibody (Santa Cruz, San Diego CA, USA) diluted 1:1000 in PBS. This antibody recognizes both native and mutated p53. Slides were washed 30 min later and matched with anti-mouse alkaline phosphatases conjugated antibody, and finally stained as described above. The evaluation of p53 status was performed as described above for MnSOD, and giving a negative value (N) for either no staining or the staining of few, sparse cells, and positive (P) for presence of immunohisochemical staining on the slice.

### RESULTS

### MnSOD content is an independent prognostic factor

30 patients affected by glioblastoma were stratified according to age (≤ 55, 13 patients; ≥ 56, 17 patients), sex (15 male and 15 female), post-surgery residual disease (≤ 10%, 6 patients; ≥ 10%, 24 patients), Karnofski Performance status (80–90, 8 patients; 60–70, 22 patients) and MnSOD levels as established by immunohistology and/or Western blot (Low levels (group I and II of Landriscina et al (1996)), 13 patients; High levels (group III and IV of Landriscina et al (1996)) 17 patients). The clinical characteristics of each patient are listed in Table 1. The ratio between LS and HS was 43% and 57%, thus slightly correcting our previous work reporting a 30/70 ratio on a smaller sample. The whole group of patients show a median survival time of 8.0 months, with Brookmeyer-Crowley 95% confidence for median survival time between 5.0 and 12.0 months. These data are in agreement with the expected survival, according to current literature (Bigner et al, 1998a). Univariate analysis of the survival of the patients was performed, and the distribution of survival times are shown in Figure 1. Confirming literature data, KPS and post-surgery residual

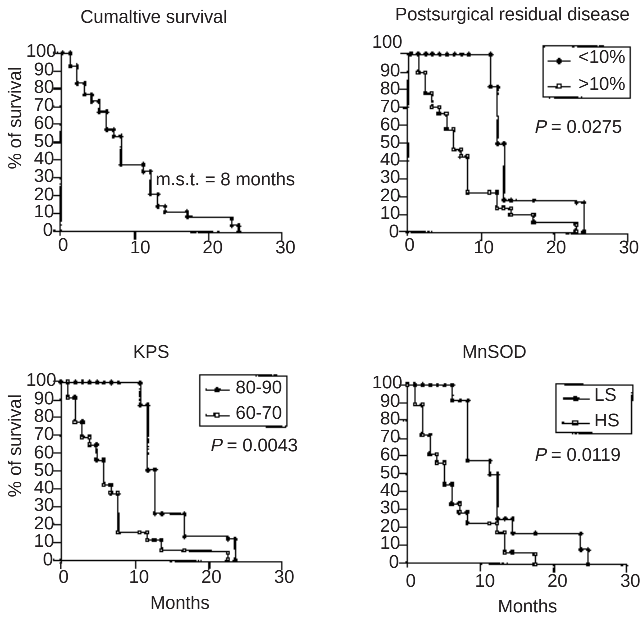

**Figure 1** MnSOD, KPS and postsurgical residual disease are prognostic factors for glioblastoma. Cumulative survival and univariate analysis of distribution of survival times of glioblastomas are shown according to postsurgical residual disease, KPS and MnSOD content. Patients were grouped as described in the text in the materials and methods section. The inclusion criteria for each group and the Mantel-Cox $P$ values for each prognostic factor are reported in the figure

© 2001 Cancer Research Campaign

**Table 2** Correlation between MnSOD level and p53

| Patient | Tumour | MnSOD (LS or HS)[a] | p53[b] |
|---|---|---|---|
| 1 | Grade II astocytoma | LS | N |
| 2 | Grade II astocytoma | LS | N |
| 3 | Grade II astocytoma | LS | N |
| 4 | Grade II astocytoma | LS | N |
| 5 | Grade III astrocytoma | HS | P |
| 6 | Grade III astrocytoma | LS | N |
| 7 | Grade III astrocytoma | LS | N |
| 8 | Grade III astrocytoma | LS | N |
| 9 | Glioblastoma | LS | N |
| 10 | Glioblastoma | HS | P |
| 11 | Glioblastoma | LS | N |
| 12 | Glioblastoma | HS | P |
| 13 | Glioblastoma | LS | N |
| 14 | Glioblastoma | LS | N |
| 15 | Glioblastoma | HS | P |
| 16 | Glioblastoma | HS | P |
| 17 | Glioblastoma | LS | N |

[a]LS and HS are described under Material and Methods. [b]N = negative; P = positive.

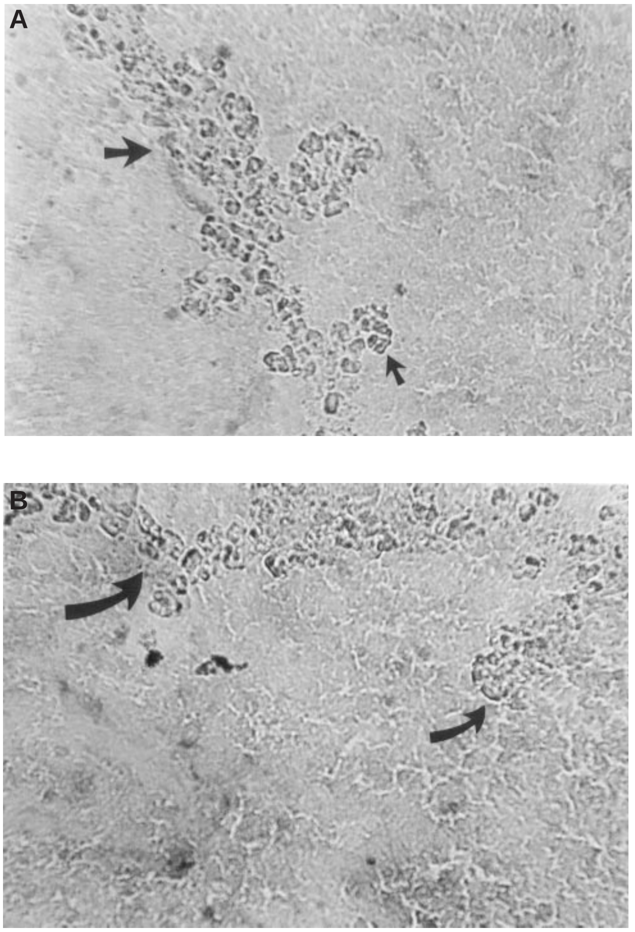

**Figure 2** Immunostaining for MnSOD (**A**) and p53 )(**B**) in adjacent slices of tumour sample from patient 10 of Table 2. The arrows indicate positively stained tumour cells infiltrating brain parenchyma

**Table 3** Grouping variables examined for significance in survival analysis

| Covariates | Univariate *P* value | Multivariate | | | |
|---|---|---|---|---|---|
| | | *P* value | Coefficient | SE | RR |
| Age (> 55/ ≤ 55) | 0.6365 | | | | |
| KPI (< 80/ ≥ 80) | 0.0043 | 0.000 | 1.9039 | 0.5588 | 6.7181 |
| MnSOD (HS/LS) | 0.0119 | 0.006 | 1.6312 | 0.4910 | 5.1100 |
| Surgery (> 90 /≤ 90) | 0.0275 | 0.5681 | | | |
| Sex (M/F) | 0.3881 | | | | |

disease were significantly associated with the clinical outcome. Thus, patients having 80–90 KPS showed a median of survival time of 12.0 months (Brookmeyer-Crowley 95% confidence interval 12.0–13.0, with mean survival time of 14.2 months) as compared to those patients that had 60–70 KPS, whose median survival time was 6.0 months (Brookmeyer-Crowley 95% confidence interval 3.0–7.0, with mean survival time of 6.4 months). The difference between the two groups was statistically significant (Mantel-Cox $P = 0.0043$). On the other hand, patients in which surgery was able to remove > 90% of tumour mass had a median survival of 12.0 months (the Brookmeyer-Crowley 95% confidence interval could not be determined; mean survival 14.2 months), while the group whose surgery was not complete displayed a median for survival time of 6.0 months (Brookmeyer-Crowley 95% confidence interval 4.0–7.0; mean survival of 7.1 months). As said above, the difference among these two groups is statistically significant (Mantel-Cox $P = 0.0275$). These observations confirm literature data (Devaux et al, 1993; Iacoangeli et al, 1993; Kowalczuk et al, 1997), and ensure that the tumour population we are dealing with is representative of the general population of patients suffering from brain tumour that are in the conditions of undergoing surgery.

When the survival of these patients is stratified according to MnSOD level in the tumour, a strong link of these levels and prognosis can be observed. HS patients show a median survival time of 5.0 months (95% confidence 3.0–6.0 months; mean survival 6.3 months), while LS patients have a median survival time of 12.0 months (95% confidence 8.0–12.0 months; mean survival 13.0 months). These differences were significant, with Mantel-Cox $P$ value = 0.0119.

As a next step, a multivariate analysis was performed between MnSOD, KPS and Postsurgical Residual Disease (see Table 3). Using this statistical analysis, the presence or absence of residual disease >10% is no longer significant (Cox multivariate model $P = 0.5681$). This effect may be due to lack of balance between the two groups. On the other hand, both MnSOD levels and KPS behave as independent prognostic factors (MnSOD rr = 5.11, $P = 0.006$; KPS rr = 6.72, $P < 0.001$). While these data confirm previous observations for KPS, the independent prognostic value of MnSOD content is an entirely new point. Thus, the difference in MnSOD content is able to describe two distinct groups of glioblastomas: a group that has low levels of MnSOD and that has a relatively better prognosis, and a group that has a high level of MnSOD and is characterized by a more severe prognosis.

 © 2001 Cancer Research Campaign

### p53 is always in cytosolic (inactivated) form when MnSOD is high

To assess to some extent the upstream mechanisms that sustain the increase in MnSOD content in brain neuroepithelial tumours, we next studied the expression of p53 in a series of 17 astrocytomas of various grading. An example of immunohistology of adjacent slices for MnSOD and p53 is shown in Figure 2, where tumour cells infiltrating the brain parenchima are positively stained for MnSOD (2A) and cytosolic p53 (2B). The sum of results of these experiments is reported in Table 2. As it is shown, in all tested astrocytomas, high MnSOD content is associated with cytoplasmic accumulation of p53. Vice versa, samples that showed low levels of MnSOD were negative for staining with p53-specific antibody. In our previous work we also reported that in few cases (that did not infringe the high statistical correlation that showed a $P < 0.001$) the Western blot and the immunohistological detection of MnSOD were not coincident. In these cases the p53 immunostaining was coincident with the immunostaining and not with the Western blot for MnSOD. When the correlation between MnSOD level (detected by immunohistology) and cytosolic accumulation of p53 was analysed with Fisher test for small numbers a $P < 0.000$ can be found. However, a caveat has to be raised due to the small absolute number of observation. As discussed below, this observation strengthens the above mentioned proposal that MnSOD can be down-regulated by active p53.

### DISCUSSION

The possibility to study a relatively homogeneous group of tumours for the correlation between amount of MnSOD and prognosis has given us an opportunity to start addressing the issue of the role of (at least one) anti-oxidant enzyme and tumorigenesis. Thus we demonstrate that the glioblastomas can be divided into two distinct groups on the basis of their content of MnSOD, and that this content is able to divide them into tumour having 'better' (low MnSOD) or 'worse' (high MnSOD) prognosis. This observation has both biological and clinical relevance.

As a first point, this observation allows us to state that the protumoral action of this enzyme is possibly more relevant than its anti-tumoral properties, in this type of tumours. However, given the tight relationship that we observed between increase of MnSOD and 'inactive status' of p53, it may be debatable whether the higher aggressiveness of tumour is actually linked to MnSOD or rather to p53, with MnSOD being an epiphenomenon. From this point of view, it gains relevance in our recent report that over-expression of MnSOD increases growth capacity of HeLa cells under serum starvation (Palazzotti et al, 1999). In this culture condition, an overproduction of ROS can be observed in wild-type HeLa cells that are not able to up-regulate their MnSOD content, and that stop proliferating and become bound to cell death. MnSOD over-production alone can rescue cells from cell death and allows them to keep on with proliferation. According to this study, we also observed that HT29 cells, that are able to up-regulate their MnSOD content, are much less sensitive to serum starvation.

In order for MnSOD to confer a selective advantage to tumour cells, it is necessary that overexpression of MnSOD as protein occurs together with increase of its enzyme activity. We therefore tested MnSOD activity of 5 glioblastomas in gel (following the method published in Palazzotti et al (1999), and we observed that it varied according to immune staining (G. Pani, unpublished observation). These data support the hypothesis that it is MnSOD that helps cells to escape cell death, to survive and to proliferate when stress conditions such as growth factors deprivation or secretion of apoptogenic cytokines occur (Mattson et al, 1997; Jayanthi et al, 1999; Shatrov et al, 2000). In an in vivo model that has offered a similar approach, it has been observed that the ischaemia-reperfusion killed colon cancer cells characterized by low metastatic potential by means of the generation of nitrogen and oxygen radicals from hepatic sinusoid endothelial cells, and that the addition of superoxide dismutase could overcome this effect (Jessup et al, 1999).

Looking at this set of data it is tempting to speculate that ROS scavenging is an advantage for tumour cells. However, the general view of literature information rather suggests that the stage of tumour progression (Okada et al, 1999), the absolute amount of ROS produced (Sung et al, 1999), the redox status of the cell and its ability to regulate the level of pro- or anti-oxidant enzymes are all together factors that will dictate the pro-or anti-tumoral effect of oxidative stress and, conversely of oxy-scavenger enzymes. Thus, it is possible that some tumour types gain advantage from the early ROS-induced DNA modification, while others rather benefit from the possibility to escape the induction of apoptosis.

As it was stated earlier, AP-1 and NF-kappa B on the positive side and p53 on the negative one have been suggested as being the major nuclear factors involved in MnSOD regulation. Our results strongly support this view. Tumours displaying high levels of MnSOD have always cytoplasmic accumulation of p53.

With the help of MnSOD we actually describe two groups of glioblastomas that differ both as for biological mechanisms of progression and as for clinical outcome. A bipartition of an otherwise homogeneous group of tumours was suggested several years ago, on the basis of histological observations that described one group of glioblastomas as being the evolution of a lower graded astrocytoma and a second one that arouses directly as a grade IV tumour (Scherer, 1938, 1940; Bigner et al, 1998b). Here we describe two biologically different groups. In the first group cytoplasmic sequestration of p53 (possibly associated with increase of signalling from membrane receptor through MAPK and AP-1 (Landriscina, unpublished observations)) leads to over-expression of MnSOD. As said above, this enzyme may offer a selective advantage in both proliferation and escape from apoptosis upon stress conditions, thus conferring a more aggressive phenotype to brain tumours. In the second group, MnSOD fails to be up-regulated and tumours show a slower progression. Further studies will be needed to establish whether the two histologically defined subgroups of glioblastomas are actually coincident with the two biologically different subgroups. In any case, the use of MnSOD as a marker may help in the future the clinical management of glioblastomas and in defining better therapeutic strategies.

### ACKNOWLEDGEMENTS

Grant Sponsor: MURST-CNR Biotechnology Program L. 95/95; Grant Nr..99.00209.PF31.

### REFERENCES

Amoroso S, Gioielli A, Cataldi M, Di Renzo G and Annunziato L (1999) In the neuronal cell line SH-SY5Y, oxidative stress-induced free radical overproduction causes cell death without any participation of intracellular $Ca^{(2+)}$ increase. *Biochem Biophys Acta* **1452**: 151–160

Bigner DD, Mclendon RE and Bruner JM (1998a) Russel and Rubinstein's Pathology of tumours of the nervous system. pp. 445–446, Arnold Edt: London

*© 2001 Cancer Research Campaign* 

Bigner DD, Mclendon RE and Bruner JM (1998b) Russel and Rubinstein's Pathology of tumours of the nervous system p. 452, Arnold Edt: London

Borrello S and Demple B (1997) NF kappa B-independent trascriptional induction of the human manganous superoxide dismutase gene. *Arch Biochem Biophys* **348**: 289–294

Bravard A, Hoffschir F, Sabatier L, Ricoul M, Pinton A, Cassingena RE, Estrade S, Luccioni C and Dutrillaux D (1992a) Early superoxide dismutase alteration during SV40 transformation of human fibroblast. *Int J Cancer* **52**: 797–801

Bravard A, Sabatier L, Hoffschir F, Ricoul M, Luccioni C and Dutrillaux D (1992b). SOD2: a new type of tumour suppressor gene? *Int J Cancer* **51**: 476–480

Bravard A, Petridis F and Luccioni C (1999) Modulation of antioxidant enzymes p21WAF1 and p53 expression during proliferation and differentiation of human melanoma cell lines. *Free Rad Biol Med* **26**: 1027–1033

Cox DR (1972). Regression model and life table. *J R Stat Soc* **34**: 187–220

Devaux BC, O'Fallon JR and Kelly PJ (1993) Resection, biopsy, and survival in malignant glial neoplasm: A retrospective study of clinical parameters, therapy, and outcome. *J Neurosur* **78**: 767–775

Galeotti T, Wohlrab H, Borrello S and De Leo ME (1989) Messenger RNA for manganese and copper/zinc superoxide dismutases in hepatomas: correlation with degree of differentiation. *Biochem Biophys Res Commmun* **165**: 581–589

Green DR and Reed JC (1998). Mitochondria and apoptosis. *Science* **281**: 1309–1312

Iacoangeli M, Rosselli R, Prezioso A, Scerrati M and Rossi GF (1993) Staging of supratentorial hemispheric glioma using tumour extension, histopathological grade, and extent of surgical resection. *Bri J Surg* **80**: 1130–1133

Ishikawa M, Yaginuma Y, Hayashi H, Shimizu T, Endo Y and Taniguchi N (1990) Reactivity of a monoclonal antibody to manganese superoxide dismutase with human ovarian carcinomas. *Cancer Res* **50**: 2538–2542

Janssen AM, Bosman CB, Kruidenier L, Griffioen G, Lamers CB Van Krieken JH, Van De Velde CJ and Verspaget HW (1999) Superoxide dismutases in the human colorectal cancer sequence. *J Cancer Res Clin Oncol* **125**: 327–335

Jayanthi S, Ordonez S, Mccoy MT and Cadet JL (1999) Dual mechanism of Fas-induced cell death in neuroglioma cells: a role for reactive oxygen species. *Brain Res Mol Brain Res* **72**: 158–165

Jessup JM, Battle P, Waller H, Edminston KH, Stolz DB, Watkins SC, Locker J and Skena K (1999) Reactive nitrogen and oxygen radicals formed during hepatic ischemia-reperfusion kill weakly metastatic colorectal cancer cells. *Cancer Res* **59**: 1825–1829

Kahlos K, Anttilia S, Asikainen T, Kinnula K, Raivio Ko, Mattson K, Linnainmaa K and Kinnula VL (1998) Manganese superoxide dismutase in healthy human pleura mesothelium and in malignant pleural mesothelioma. *Am J Respir Cell Mol Biol* **18**: 570–580

Kaplan EL And Meyer P (1958) Nonparametric estimation from incomplete observation. *J Am Stat Assoc* **53**: 457–481

Kawamura N, Suzuki K, Ishikawa M, Iizuka S, Miyake M, Mino M and Taniguchi N (1992) High levels of Mn-superoxide dismutase in serum of patients with neuroblastoma and in human neuroblastoma cell lines. *Free Rad Biol Med* **12**: 281–286

Kinningham KK, Oberley TD, Lin S, Mattingly CA and St Clair DK (1999) Over-expression of manganese superoxide dismutase protects against mitochondrial-initiated poly(ADP-ribose) polymerase-mediated cell death. *FASEB J* **13**: 1601–1610

Kowalczuk A, Macdonald RL, Amidei C, Dohrmann G 3rd, Erickson RK, Hekmatpanah J, Krauss S, Krishnasamy S, Masters G, Mullan SF, Mundt AJ, Sweeney P, Vokes EE, Weir BK and Wollman RL (1997) Quantitative imaging study of extent of surgical resection and prognosis of malignant astrocytomas. *Neurosurgery* **41**: 1028–1038

Lam EW, Zwacka R, Seftor EA, Nieva DR, Davidson BL, Engelhardt JF, Hendrix MJ and Oberley LW (1999). Effects of antioxidant enzymes overexpression on the invasive phenotype of hamster cheek pouch carcinoma cells. *Free Rad Biol Med* **27**: 572–579

Landriscina M, Remiddi F, Ria F, Palazzotti B, De Leo ME, Iacoangeli M, Rosselli R, Scerrati M and Galeotti T (1996) The level of MnSOD is directly correlated with grade of brain tumours of neuroepithelial origin. *Br J Cancer* **74**: 1877–1885

Li N, Oberley TD, Oberley LW and Zhong W (1998) Overexpression of manganese superoxide dismutase in DU145 human prostate carcinoma cells has multiple effects on cell phenotype. *Prostate* **35**: 221–233

Mattson MP, Goodman Y, Luo H, Fu W and Furukawa K (1997) Activation of NF-kappaB protects hippocampal neurons against oxidative stress-induced apoptosis: evidence for induction of manganese superoxide dismutase and suppression of peroxynitrite production and protein tyrosine nitration. *J Neurosci Res* **49**: 681–697

Nakano T, Oka K and Taniguchi N (1996) Manganese Superoxide Dismutase expression correlates with p53 status and local recurrence of cervical carcinoma treated with radiation therapy. *Cancer Res* **56**: 2771–2775

Okada F, Nakai K, Kobayashi T, Shibata T, Tagami S, Kawakami Y, *et al* (1999). Inflammatory cell-mediated tumour progression and minisatellite mutation correlate with the decrease of antioxidative enzymes in murine fibrosarcoma cells. *Br J Cancer* **79**: 377–385

Palazzotti B, Pani G, Colavitti R, De Leo ME, Bedogni B, Borrello S and Galeotti T (1999) Increased growth capacity of cervical-carcinoma cells over-expressing manganous superoxide dismutase. *Int J Cancer* **82**: 145–150

Pani G, Bedogni B, Anzevino R, Colavitti R, Palazzotti B, Borrello S and Galeotti T (2000) Deregulated manganese superoxide dismutase expression and resistance to oxidative injury in p53-deficient cells. *Cancer Res* **60**: 4654–4660

Polyak K, Xia Y, Zweier JL, Kinzler KW and Vogelstein B (1997) A model for p53-induced apoptosis. *Nature* **389**: 300–305

Ria F, Landriscina M and Galeotti T (1993) Preparation of a monoclonal antibody against rat MnSOD, using a COOH-terminal peptide. *Biochem Biophys Res Commun* **195**: 697–703

Sarto C, Frutiger S, Cappellano F, Sanchez JC, Doro G, Catanzaro F, Huges JH, Hochstrasser DF and Mocarelli P (1999). Modified expression of plasma glutathione peroxidase and manganese superoxide dismutase in human renal cell carcinoma. *Electrophoresis* **20**: 3458–3466

Scherer HJ (1938) Structural development in gliomas. *Am J Cancer* **34**: 333–351

Scherer HJ (1940) Cerebral astrocytomas and their derivatives. *Am J Cancer* **40**: 159–198

Shatrov VA, Ameyar M, Bouquet C, Cai Z, Stancou R, Haddada H and Chouaib S (2000) Adenovirus mediated wild-type-p53-gene expression sensitizes TNF-resistant tumor cells to TNF-induced cytotoxicity by altering the cellular redox state. *Int J Cancer* **85**: 93–97

Sun Y. (1990) Free radicals, antioxidant enzymes and carcinogenesis. *Free Rad Biol Med* **8**: 583–599

Sung YJ, Juan CC, Lee HC, Yin PH, Chi CW, Ku HH, Li AF, Wei YH and Tsay HJ (1999) Oxidative stress is insignificant in N1S1-trasplanted hepatoma despite markedly declined activities of the antioxidant enzymes. *Oncol Rep* **6**: 1313–1319

Xu Y, Kinningham KK, Devalaraja MN, Yeh CC. Majima H, Kasarskis EJ And St Clair DK (1999) An intronic NF-kappaB element is essential for induction of the human manganese superoxide dismutase gene by tumour necrosis factor-alpha and interleukin 1 beta. *DNA Cell Biol* **18**: 709–722

Yoshii Y, Saito A, Zhao DW and Nose T (1999) Copper/zinc superoxide dismutase, nuclear DNA content, and progression in human gliomas. *J Neurooncol*., **42**: 103–108

 *© 2001 Cancer Research Campaign*

## References

[PDF_00544] Amoroso S., Gioielli A., Cataldi M., Di Renzo G., Annunziato L. (1999). In the neuronal cell line SH-SY5Y, oxidative stress-induced free radical overproduction causes cell death without any participation of intracellular Ca(2+) increase.. Biochim Biophys Acta.

[PDF_00554] Borrello S., Demple B. (1997). NF kappa B-independent transcriptional induction of the human manganous superoxide dismutase gene.. Arch Biochem Biophys.

[PDF_00557] Bravard A., Hoffschir F., Sabatier L., Ricoul M., Pinton A., Cassingena R., Estrade S., Luccioni C., Dutrillaux B. (1992). Early superoxide dismutase alterations during SV40-transformation of human fibroblasts.. Int J Cancer.

[PDF_00562] Bravard A., Petridis F., Luccioni C. (1999). Modulation of antioxidant enzymes p21WAF1 and p53 expression during proliferation and differentiation of human melanoma cell lines.. Free Radic Biol Med.

[PDF_00560] Bravard A., Sabatier L., Hoffschir F., Ricoul M., Luccioni C., Dutrillaux B. (1992). SOD2: a new type of tumor-suppressor gene?. Int J Cancer.

[PDF_00566] Devaux B. C., O'Fallon J. R., Kelly P. J. (1993). Resection, biopsy, and survival in malignant glial neoplasms. A retrospective study of clinical parameters, therapy, and outcome.. J Neurosurg.

[PDF_00569] Galeotti T., Wohlrab H., Borrello S., De Leo M. E. (1989). Messenger RNA for manganese and copper-zinc superoxide dismutases in hepatomas: correlation with degree of differentiation.. Biochem Biophys Res Commun.

[PDF_00572] Green D. R., Reed J. C. (1998). Mitochondria and apoptosis.. Science.

[PDF_00574] Iacoangeli M., Roselli R., Prezioso A., Scerrati M., Rossi G. F. (1993). Staging of supratentorial hemispheric glioma using tumour extension, histopathological grade and extent of surgical resection.. Br J Surg.

[PDF_00577] Ishikawa M., Yaginuma Y., Hayashi H., Shimizu T., Endo Y., Taniguchi N. (1990). Reactivity of a monoclonal antibody to manganese superoxide dismutase with human ovarian carcinoma.. Cancer Res.

[PDF_00581] Janssen A. M., Bosman C. B., Kruidenier L., Griffioen G., Lamers C. B., van Krieken J. H., van de Velde C. J., Verspaget H. W. (1999). Superoxide dismutases in the human colorectal cancer sequence.. J Cancer Res Clin Oncol.

[PDF_00583] Jayanthi S., Ordonez S., McCoy M. T., Cadet J. L. (1999). Dual mechanism of Fas-induced cell death in neuroglioma cells: a role for reactive oxygen species.. Brain Res Mol Brain Res.

[PDF_00586] Jessup J. M., Battle P., Waller H., Edmiston K. H., Stolz D. B., Watkins S. C., Locker J., Skena K. (1999). Reactive nitrogen and oxygen radicals formed during hepatic ischemia-reperfusion kill weakly metastatic colorectal cancer cells.. Cancer Res.

[PDF_00590] Kahlos K., Anttila S., Asikainen T., Kinnula K., Raivio K. O., Mattson K., Linnainmaa K., Kinnula V. L. (1998). Manganese superoxide dismutase in healthy human pleural mesothelium and in malignant pleural mesothelioma.. Am J Respir Cell Mol Biol.

[PDF_00596] Kawamura N., Suzuki K., Ishikawa M., Iizuka S., Miyake M., Mino M., Taniguchi N. (1992). High levels of Mn-superoxide dismutase in serum of patients with neuroblastoma and in human neuroblastoma cell lines.. Free Radic Biol Med.

[PDF_00600] Kiningham K. K., Oberley T. D., Lin S., Mattingly C. A., St Clair D. K. (1999). Overexpression of manganese superoxide dismutase protects against mitochondrial-initiated poly(ADP-ribose) polymerase-mediated cell death.. FASEB J.

[PDF_00605] Kowalczuk A., Macdonald R. L., Amidei C., Dohrmann G., Erickson R. K., Hekmatpanah J., Krauss S., Krishnasamy S., Masters G., Mullan S. F. (1997). Quantitative imaging study of extent of surgical resection and prognosis of malignant astrocytomas.. Neurosurgery.

[PDF_00609] Lam E. W., Zwacka R., Seftor E. A., Nieva D. R., Davidson B. L., Engelhardt J. F., Hendrix M. J., Oberley L. W. (1999). Effects of antioxidant enzyme overexpression on the invasive phenotype of hamster cheek pouch carcinoma cells.. Free Radic Biol Med.

[PDF_00613] Landriscina M., Remiddi F., Ria F., Palazzotti B., De Leo M. E., Iacoangeli M., Rosselli R., Scerrati M., Galeotti T. (1996). The level of MnSOD is directly correlated with grade of brain tumours of neuroepithelial origin.. Br J Cancer.

[PDF_00616] Li N., Oberley T. D., Oberley L. W., Zhong W. (1998). Overexpression of manganese superoxide dismutase in DU145 human prostate carcinoma cells has multiple effects on cell phenotype.. Prostate.

[PDF_00619] Mattson M. P., Goodman Y., Luo H., Fu W., Furukawa K. (1997). Activation of NF-kappaB protects hippocampal neurons against oxidative stress-induced apoptosis: evidence for induction of manganese superoxide dismutase and suppression of peroxynitrite production and protein tyrosine nitration.. J Neurosci Res.

[PDF_00624] Nakano T., Oka K., Taniguchi N. (1996). Manganese superoxide dismutase expression correlates with p53 status and local recurrence of cervical carcinoma treated with radiation therapy.. Cancer Res.

[PDF_00627] Okada F., Nakai K., Kobayashi T., Shibata T., Tagami S., Kawakami Y., Kitazawa T., Kominami R., Yoshimura S., Suzuki K. (1999). Inflammatory cell-mediated tumour progression and minisatellite mutation correlate with the decrease of antioxidative enzymes in murine fibrosarcoma cells.. Br J Cancer.

[PDF_00631] Palazzotti B., Pani G., Colavitti R., De Leo M. E., Bedogni B., Borrello S., Galeotti T. (1999). Increased growth capacity of cervical-carcinoma cells over-expressing manganous superoxide dismutase.. Int J Cancer.

[PDF_00634] Pani G., Bedogni B., Anzevino R., Colavitti R., Palazzotti B., Borrello S., Galeotti T. (2000). Deregulated manganese superoxide dismutase expression and resistance to oxidative injury in p53-deficient cells.. Cancer Res.

[PDF_00637] Polyak K., Xia Y., Zweier J. L., Kinzler K. W., Vogelstein B. (1997). A model for p53-induced apoptosis.. Nature.

[PDF_00639] Ria F., Landriscina M., Galeotti T. (1993). Preparation of a monoclonal antibody against rat MnSOD, using a COOH-terminal peptide.. Biochem Biophys Res Commun.

[PDF_00642] Sarto C., Frutiger S., Cappellano F., Sanchez J. C., Doro G., Catanzaro F., Hughes G. J., Hochstrasser D. F., Mocarelli P. (1999). Modified expression of plasma glutathione peroxidase and manganese superoxide dismutase in human renal cell carcinoma.. Electrophoresis.

[PDF_00649] Shatrov V. A., Ameyar M., Bouquet C., Cai Z., Stancou R., Haddada H., Chouaib S. (2000). Adenovirus-mediated wild-type-p53-gene expression sensitizes TNF-resistant tumor cells to TNF-induced cytotoxicity by altering the cellular redox state.. Int J Cancer.

[PDF_00653] Sun Y. (1990). Free radicals, antioxidant enzymes, and carcinogenesis.. Free Radic Biol Med.

[PDF_00655] Sung Y. J., Juan C. C., Lee H. C., Yin P. H., Chi C. W., Ku H. H., Li A. F., Wei Y. H., Tsay H. J. (1999). Oxidative stress is insignificant in N1S1-transplanted hepatoma despite markedly declined activities of the antioxidant enzymes.. Oncol Rep.

[PDF_00660] Xu Y., Kiningham K. K., Devalaraja M. N., Yeh C. C., Majima H., Kasarskis E. J., St Clair D. K. (1999). An intronic NF-kappaB element is essential for induction of the human manganese superoxide dismutase gene by tumor necrosis factor-alpha and interleukin-1beta.. DNA Cell Biol.

[PDF_00663] Yoshii Y., Saito A., Zhao D. W., Nose T. (1999). Copper/zinc superoxide dismutase, nuclear DNA content, and progression in human gliomas.. J Neurooncol.

